# Correlation of Insulin Resistance with Anthropometric Measures and Blood Pressure in Adolescents

**DOI:** 10.5935/abc.20160041

**Published:** 2016-04

**Authors:** Polyana Resende Silva de Morais, Ana Luiza Lima Sousa, Thiago de Souza Veiga Jardim, Flávia Miquetichuc Nogueira Nascente, Karla Lorena Mendonça, Thaís Inácio Rolim Povoa, Carolina de Souza Carneiro, Vanessa Roriz Ferreira, Weimar Kunz Sebba Barroso de Souza, Paulo César Brandão Veiga Jardim

**Affiliations:** Liga de Hipertensão Arterial/Hospital das Clínicas da Universidade Federal de Goiás (UFG), GO - Brazil

**Keywords:** Blood Pressure, Body Mass Index, Insulin Resistance, Anthropometry, Adolescent

## Abstract

**Background:**

Blood pressure is directly related to body mass index, and individuals with increased waist circumference have higher risk of developing hypertension, insulin resistance, and other metabolic changes, since adolescence.

**Objective:**

to evaluate the correlation of blood pressure with insulin resistance, waist circumference and body mass index in adolescents.

**Methods:**

Cross-section study on a representative sample of adolescent students. One group of adolescents with altered blood pressure detected by casual blood pressure and/or home blood pressure monitoring (blood pressure > 90^th^ percentile) and one group of normotensive adolescents were studied. Body mass index, waist circumference were measured, and fasting glucose and plasma insulin levels were determined, using the HOMA-IR index to identify insulin resistance.

**Results:**

A total of 162 adolescents (35 with normal blood pressure and 127 with altered blood pressure) were studied; 61% (n = 99) of them were boys and the mean age was 14.9 ± 1.62 years. Thirty-eight (23.5%) adolescents had altered HOMA-IR. The group with altered blood pressure had higher values of waist circumference, body mass index and HOMA-IR (p<0.05). Waist circumference was higher among boys in both groups (p<0.05) and girls with altered blood pressure had higher HOMA-IR than boys (p<0.05). There was a significant moderate correlation between body mass index and HOMA-IR in the group with altered blood pressure (ρ = 0.394; p < 0.001), and such correlation was stronger than in the normotensive group. There was also a significant moderate correlation between waist circumference and HOMA-IR in both groups (ρ = 0.345; p < 0.05). Logistic regression showed that HOMA-IR was as predictor of altered blood pressure (odds ratio - OR = 2.0; p = 0.001).

**Conclusion:**

There was a significant association of insulin resistance with blood pressure and the impact of insulin resistance on blood pressure since childhood. The correlation and association between markers of cardiovascular diseases was more pronounced in adolescents with altered blood pressure, suggesting that primary prevention strategies for cardiovascular risk factors should be early implemented in childhood and adolescence.

## Introduction

Hypertension is one of the main risk factors for cardiovascular diseases, which are the main cause of deaths in Brazil and in the world.^1-3^ In the last decade, high blood pressure levels have been identified in children and adolescents.^1,4-6^

Obesity is highlighted as one of the important risk factors for hypertension, and it reaches epidemic proportions in many parts of the world.^7-9^ Body fat mass is associated with profound changes in physiological functions, including from alterations in blood volume homeostasis to changes in left ventricular function. It is also indicated as a potential causal link between hypertension and insulin resistance (IR), among other metabolic changes.^8,10^ It is estimated that 20%-30% of overweight / obese children and adolescents have hypertension.^11,12^

Body composition is one of the main determinants of high blood pressure in childhood and adolescence. There is a direct relationship between weight, body mass index (BMI) and hypertension, particularly in the second decade of life.^13^

The strong association between high blood pressure and excessive weight has led to an increase in the prevalence of hypertension among children and adolescents.^8^ Waist circumference (WC) has a good predictive value for abdominal obesity-related diseases in adolescents, and increased WC values have been considered as a significant risk factor for IR and cardiovascular diseases.^14^

IR is also considered a risk marker for cardiovascular disease, and is associated with several metabolic changes related to, but not exclusively associated with obesity or type 2 diabetes.^15,16^ For decades, abdominal fat has been associated with hyperinsulinemia, which is a predictor of hypertension and dyslipidemias.^9,17^

The homeostasis model assessment as an index of IR (HOMA-IR) is a rapid, easy, low-cost method, which has been used as an alternative approach for IR diagnosis.^19^

There are no studies in Brazil correlating IR and blood pressure in adolescents aged over 12 years, and few studies have evaluated the correlation between IR and anthropometric variables in this population. The aim of this study was to evaluate the correlation between IR, WC, BMI and blood pressure in adolescents, and the behavior of these variables by sex.

## Methods

This was a cross-sectional study, part of the original project CorAdo (*Coração de Adolescente*, Adolescent's heart). The study was approved by the local Ethics Committee (protocol: 017/2010), and conducted in a capital city of Brazil in 2012. The sample was representative of adolescent students, enrolled in the city's (public or private) schools.

In the initial sample of 1,025 adolescents, stratified by sex, anthropometric measurements were performed, as well as casual blood pressure and home blood pressure monitoring (HBPM).

WC was measured using a non-elastic measurement tape (200 cm). The cut-off points were adjusted by sex and age, and the 90th percentile was set as indicator of metabolic changes.^20^

Body weight was measured to the nearest 0.1 kg using an electronic, portable scale (Kratos^®^, 150 kg capacity), calibrated by the National Institute of Metrology, Quality and Technology (Inmetro). Height was measured to the nearest 0.1 cm using a wall-mounted stadiometer (Secca^®^). All measurements were performed following the World Health Organization guidelines (WHO). ^21^

BMI was calculated by dividing body weight (kilograms) by the square of the height (meters).^22^ The adolescents were classified into obese or overweight based on WHO BMI cut-off points for age and sex (WHO).^23^

Casual blood pressure and HBPM were measured using Omron HEM-705CP semi-automatic blood pressure monitors and different sizes of cuffs, in accordance to the *4^th^ Task Force's* recommendations.^24^

Four measures of casual blood pressure were taken, the first two measures when the blood pressure monitor was handed to patients, and the other two when patients returned the monitors one week later. There was a 3-min interval between measurements. The mean of the second readings was used for analysis. Blood pressure percentile was calculated using the formulas proposed by the 4^th^ Task Force, using the MeDCal 3000 software.

Adolescents and caregivers were instructed in the use of HBPM, to take four blood pressure measures, two in the morning (between 7h and 9h) and two in the afternoon (between 18h and 19h), with a 3-5 min-interval between them. One week later, participants returned the monitors, totaling 6 days of measurements.

The diagnosis of altered blood pressure (casual or HBPM) was determined according to international guidelines. Normal blood pressure was defined as having systolic pressure below the 90th percentile and blood pressure readings below 120/80 mmHg, and altered blood pressure was defined as systolic pressure greater than the 90th percentile.

Since there are no validated criteria for HBPM, we used the criteria proposed by the 4^th^ Task Force in the study by Stergiou et al.,^25^ which suggests that both casual and HBPM measures should be similar in adolescents aged greater than 12 years.

Of the initial sample (n = 1,025), 198 (19.3%) adolescents had altered systolic and/or diastolic blood pressure in the casual measurement and/or HBPM, and composed the potential group for phase 2.

**Figure 1:**
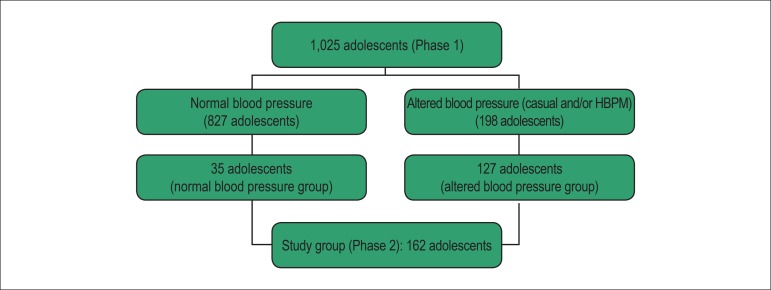
Fluxogram of sample composition for the phase 2 of the study.

For sample size calculation, an error of 5% and power of 80% were fixed, considering the number of subjects with altered blood pressure (n= 198) identified from the initial sample during phase 1 of the CorAdo study. A minimum of 127 adolescents were required, and we also included 35 adolescents with normal blood pressure (controls), who were invited to the phase 2 of the study. A total of 162 adolescents completed the study (Figure 1).

Participants' parents or caregivers signed the informed consent form before participating in the phase 2 of the study. Adolescents who met the inclusion criteria answered a questionnaire and had their blood collected.

Sexual maturation was assessed by self-assessment, using Tanner's photographs of five sexual maturation stages.^26^ Children classified as prepubertal (Tanner stage I) were withdrawn from the study.

Inclusion criteria were adolescents aged from 12 to 18 years (to be completed), enrolled in public and private schools, with altered blood pressure (by casual measurement and/or HBPM), and Tanner stage ≥ 2 (pubertal stage).

Exclusion criteria included patients with physical disabilities that hinder blood pressure measurement, self-reported chronic disease, diabetes mellitus, kidney disease or heart disease, pregnancy, and chronic use of medications that may affect blood pressure, such as antihypertensive drugs, corticosteroids, antidepressants, anxiolytics, anti-inflammatories, and oral contraceptives.

Serum glucose and plasma insulin levels were determined. The HOMA-IR index (insulin *µ*u/mL x glycemia mmol/L/22.5) was used to quantify IR, whose threshold set for adolescents is ≥ 3.16;^27^ values of glycemia (mg/dL) were multiplied by 0.05551.^28,29^

### Statistical analysis

Statistical analysis was performed using the *Statistical Package for Social Science* (SPSS) software version 20 (IBM, Chicago, IL, USA) and Epi-Info^™^. The Kolmogorov-Smirnov test was used to test the normality of the continuous variables and the Mann-Whitney U test to compare the means of the variables. Values were expressed as mean, median, standard deviation and confidence interval. A descriptive analysis of data was performed; associations between categorical variables were tested by the chi-square test, and the Spearman correlation was used to assess the association between blood pressure and BMI, WC, and HOMA-IR.

Stepwise regression was conducted, considering changes in blood pressure as dependent variable. In the bivariate analysis, variables with a p-value < 0.20 were considered predictors. The level of significance was set at p< 0.05.

## Results

A total of 162 adolescents participated in the phase 2 of the study, 127 with altered blood pressure and 35 controls. Mean age of participants was 14.9 ± 1.62 years, and 61.1% were male.

Thirty-eight adolescents (23.5%) had altered HOMA-IR, 74 (45.7%) were overweight/obese, and 17 (10.5%) had increased WC (Table 1).

**Table 1:** Anthropometric and biochemical characteristics of the study group (n = 162)

**Variables**	**n (%)**	p value*
Waist circumference		0.005
Normal	145 (89.5)	
Increased	17 (10.5)	
Body mass index		< 0.001
Normal	88 (54.3)	
Overweight	39 (24.1)	
Obese	35 (21.6)	
HOMA-IR		< 0.001
Normal	124 (76.5)	
Altered	38 (23.5)	

* Chi-square test. HOMA-IR: Homeostasis Model Assessment - Insulin Resistance.

Mean values of HOMA-IR, BMI and WC were significantly higher in the group with altered blood pressure than in controls (Table 2).

**Table 2:** Blood pressure, Homeostasis Model Assessment - Insulin Resistance (HOMA-IR) index, waist circumference (WC) and body mass index (BMI) (n = 162)

**Variables**	**Blood pressure**				p value*
	**Normal (n = 35)**		**Altered (n = 127)**		
	**Mean**	**SD**	**Mean**	**SD**	
HOMA-IR	1.8	± 1.1	2.8	± 1.7	≤ 0.001
WC, cm	71.0	± 10.0	76.5	± 11.0	0.001
BMI, kg/m^2^	21.1	± 3.7	23.8	_._8 ±	0.001

* Mann-Whitney U test. SD: standard deviation.

When variables were categorized considering the normality criteria, a significant association was found only between blood pressure and BMI (p < 0.022), with 50.4% of participants with altered blood pressure and excessive weight, and no difference in sex distribution.

HOMA-IR index and BMI were similar between sexes. Mean WC was higher among male adolescents in both groups (altered blood pressure and normotensive) (p<0.05) (Tables 3 and 4). In the group of adolescents with altered blood pressure group, HOMA-IR indexes were higher in female than in male adolescents (p<0.05) (Table 4).

**Table 3:** Relationship between Homeostasis Model Assessment - Insulin Resistance (HOMA-IR), waist circumference (WC) and body mass index (BMI) in normotensive adolescents (n = 35)

**Variables**	**Sex**				p value*
	**Male (n = 99)**		**Female (n = 63)**		
	**Mean Mean**	**SD95 % CI**	**Mean Median**	**SD95 %CI**	
HOMA-IR	1.9	1.3	1.7	0.7	0.960
	1.5	0.65-6.12	1.5	0.7-3.4	
WC, cm	74.2	11.2	65.8	4.4	0.009
	70.6	61-107	65.3	58.5-75.0	
BMI, kg/m^2^	21.5	4.0	20.4	3.1	0.511
	20.6	17.0-30.7	19.6	16.8-26.3	

* Mann-Whitney U test. SD: standard deviation; 95%CI: 95% confidence interval.

**Table 4:** Relationship between Homeostasis Model Assessment - Insulin Resistance (HOMA-IR), waist circumference (WC) and body mass index (BMI) in adolescents with altered blood pressure (n = 127)

**Variables**	**Sex**				p value*
	**Male (n = 99)**		**Female (n = 63)**		
	**Mean Median**	**SD95 %CI**	**Mean Median**	**SD95 %CI**	
HOMA-IR	2.7	1.7	3.1	1.7	0.036
	2.2	0.53-8.39	2.7	0.61-8.57	
WC, cm	78.1	10.9	74.1	10.7	0.035
	76.2	61-120	70.7	56-107	
BMI, kg/m^2^	23.8	4.1	23.7	5.7	0.248
	23.4	15.9-35.0	22.5	16.1-42.5	

* Mann-Whitney U test. SD: standard deviation; 95%CI: 95% confidence interval.

There was a direct, moderate correlation between blood pressure and HOMA-IR (ρ = 0.323; p < 0.001), and a statistically significant but weak correlation between blood pressure and BMI, and between blood pressure and WC (ρ = 0.254; p = 0.001; e ρ = 0.258; p = 0.001).

In the group analysis, stronger correlations between variables were detected, especially between BMI and HOMA-IR in the group of altered blood pressure (ρ = 0.394; p < 0.001). Similar correlations between WC and HOMA-IR were found in both groups (ρ = 0.345; p < 0.05) (Table 5).

**Table 5:** Correlation of Homeostasis Model Assessment - Insulin Resistance (HOMA-IR) index, with body mass index (BMI) and waist circumference (WC) in adolescents with normotensive adolescents (n = 35) and altered blood pressure (n=127)

**Variables**	**Normal blood pressure (n= 35)**		**Altered blood pressure (n= 127)**	
	**Spearman [Table-fn TFN5]**	p value*	**Spearman**	p value*
HOMA-IR and BMI	0.366	0.031	0.394	< 0.001
HOMA-IR and WC	0.345	0.042	0.345	< 0.001

* Spearman correlation test.

In the logistic regression analysis, blood pressure was affected only by HOMA-IR (*odds ratio* - OR = 2.0; p = 0.001).

## Discussion

In many parts of the world, the prevalence of adult diseases, considered risk factors for cardiovascular diseases, has increased in pediatric population. Few studies have investigated the correlation/association between IR and blood pressure, especially in this population.

In this study, there was a positive association between mean values of HOMA-IR index and altered blood pressure in adolescents (p < 0.001). In the Bogalusa Heart Study, also conducted on adolescents, the HOMA-IR values were higher than those observed in our study. In another study carried out in Rio de Janeiro, the authors also reported higher HOMA-IR indexes, although the study group was composed of adults rather than adolescents.^30,31^ In a pilot study conducted in Eastern Europe involving 128 children, HOMA-IR indexes were similar to our findings.^32^

The prevalence of IR in our study group was 23.5%, considering a HOMA-IR cut-off point of 3.16, proposed by Keskin et al.^27^ In Cochabamba, Bolivia, a study on 61 children and adolescents adopted^33^ a different HOMA-IR cut-off (3.5), and reported a 39.4% prevalence of IR. A higher prevalence of IR was found in children and adolescents with high systolic pressure (p < 0.05).

In this study, HOMA-IR was not correlated with changes in blood pressure by using the absolute cut-off points. However, a significant direct correlation was found between mean HOMA-IR values and changes in blood pressure percentiles (ρ = 0.323; p < 0.001). This is in accordance with a study carried out in India, involving 2,640 adolescents.^34^

Female adolescents with altered blood pressure had higher mean HOMA-IR values (p<0.05), which was not observed in the normotensive group. Previous studies^7,34^ have reported a high prevalence of altered HOMA-IR among female adolescents, which may be in part explained by differences in body fat distribution or pubertal stages as compared with boys. With respect to sexual maturation, girls may enter puberty two years earlier than boys. In the absence of other known variables, these findings suggest that girls tend to be more resistance to insulin than boys due to sex-linked genes.^35^

It is worth mentioning that previous studies have not reported differences in the mean values of HOMA-IR between sexes,^33,36^ and one study has found a higher IR among boys than girls^17^. Further studies are needed to elucidate these conflicting results.

By logistic regression, our study identified, for the first time, that adolescents with altered HOMA-IR are twice as likely to have altered blood pressure (OR = 2.0; p = 0.001)

Other variables, such as BMI and WC did not affect the chance of having altered blood pressure. This result differed from that found in a study carried out in the south of Brazil on 1,950 children and adolescents, describing a positive relationship of systolic pressure to BMI and WC.^37,38^

Some studies have reported an association between BMI and HOMA-IR, which may be explained by the increased anabolic effect of insulin and growth hormone related to the rapid somatic growth of children during puberty. This change in insulin sensitivity results from changes in body fat distribution in this period of life.^17,36^

In the present study, a significant, moderate correlation was observed between BMI and HOMA-IR (ρ = 0.394; p < 0.001, for adolescents with altered blood pressure; and ρ = 0.366; p < 0.031, for normotensive adolescents. This is in accordance with previous investigations that showed that the prevalence of IR is more than twice as high among overweight and obese children and adolescents.^36,39,40^

When analyzed by sex, we observed that male adolescents of both groups had higher mean WC, similarly to previous studies.^7,36^

In addition, we found a positive correlation between WC and HOMA-IR (ρ = 0.345; p < 0.001 for altered blood pressure group; and ρ = 0.345; p = 0.042 for normotensive group). Singh and colleagues also found a strong correlation between HOMA-IR and WC,^36^ and studies conducted in Brazil reported a significant association between WC and HOMA-IR in female adolescents.^39,41^

Our study differs from previous studies in the analysis of correlations between variables (particularly HOMA-IR and BMI) by group, i.e. between adolescents with altered blood pressure and normotensive subjects.

The study has some limitations that need to be considered. First, the lack of a comprehensive assessment of body composition including other methods such as skinfold thickness or electrical bioimpedance analysis, and second, the possible inaccuracy of the method used for assessing sexual maturation.

## Conclusion

There was a significant association of IR with blood pressure, and the impact of IR on blood pressure. The correlation and association between markers of cardiovascular diseases was more pronounced in adolescents with altered blood pressure, suggesting the need for primary prevention strategies for cardiovascular risk factors in childhood and adolescence.
